# Immunomodulatory Effects of Heme Oxygenase-1 in Kidney Disease

**DOI:** 10.3389/fmed.2021.708453

**Published:** 2021-08-24

**Authors:** Yunlong Li, Kuai Ma, Zhongyu Han, Mingxuan Chi, Xiyalatu Sai, Ping Zhu, Zhaolun Ding, Linjiang Song, Chi Liu

**Affiliations:** ^1^Guangdong Cardiovascular Institute, Guangdong Provincial People's Hospital, Guangdong Academy of Medical Sciences, Guangzhou, China; ^2^School of Medical and Life Sciences, Reproductive and Women-Children Hospital, Chengdu University of Traditional Chinese Medicine, Chengdu, China; ^3^Department of Nephrology, Osaka University Graduate School of Medicine, Osaka, Japan; ^4^Department of Emergency Surgery, Shannxi Provincial People's Hospital, Xi'an, China; ^5^Department of Nephrology, Sichuan Academy of Medical Science and Sichuan Provincial People's Hospital, University of Electronic Science and Technology of China, Chengdu, China

**Keywords:** heme oxygenase-1, kidney diseases, immune regulation, oxidative stress, carbon monoxide

## Abstract

Kidney disease is a general term for heterogeneous damage that affects the function and the structure of the kidneys. The rising incidence of kidney diseases represents a considerable burden on the healthcare system, so the development of new drugs and the identification of novel therapeutic targets are urgently needed. The pathophysiology of kidney diseases is complex and involves multiple processes, including inflammation, autophagy, cell-cycle progression, and oxidative stress. Heme oxygenase-1 (HO-1), an enzyme involved in the process of heme degradation, has attracted widespread attention in recent years due to its cytoprotective properties. As an enzyme with known anti-oxidative functions, HO-1 plays an indispensable role in the regulation of oxidative stress and is involved in the pathogenesis of several kidney diseases. Moreover, current studies have revealed that HO-1 can affect cell proliferation, cell maturation, and other metabolic processes, thereby altering the function of immune cells. Many strategies, such as the administration of HO-1-overexpressing macrophages, use of phytochemicals, and carbon monoxide-based therapies, have been developed to target HO-1 in a variety of nephropathological animal models, indicating that HO-1 is a promising protein for the treatment of kidney diseases. Here, we briefly review the effects of HO-1 induction on specific immune cell populations with the aim of exploring the potential therapeutic roles of HO-1 and designing HO-1-based therapeutic strategies for the treatment of kidney diseases.

## Introduction

Kidney disease is an umbrella term for a number of diseased states characterized by impaired kidney function and/or structure. Due to the occult onset and the difficulty in early diagnosis, a large number of patients with kidney diseases end up with kidney failure and complete loss of kidney function (1). Currently, kidney diseases are still treated by surgery, chronic dialysis, renal transplantation, and other means, but these methods are suboptimal in the complete treatment of these diseases (1). Thus, the development of new treatments and drugs to change this status remains a pressing priority (1). Several potential renal protective therapies are currently being investigated, of which enhancing the heme oxygenase (HO) system to protect renal morphology is an area of great interest.

**Figure 1:**
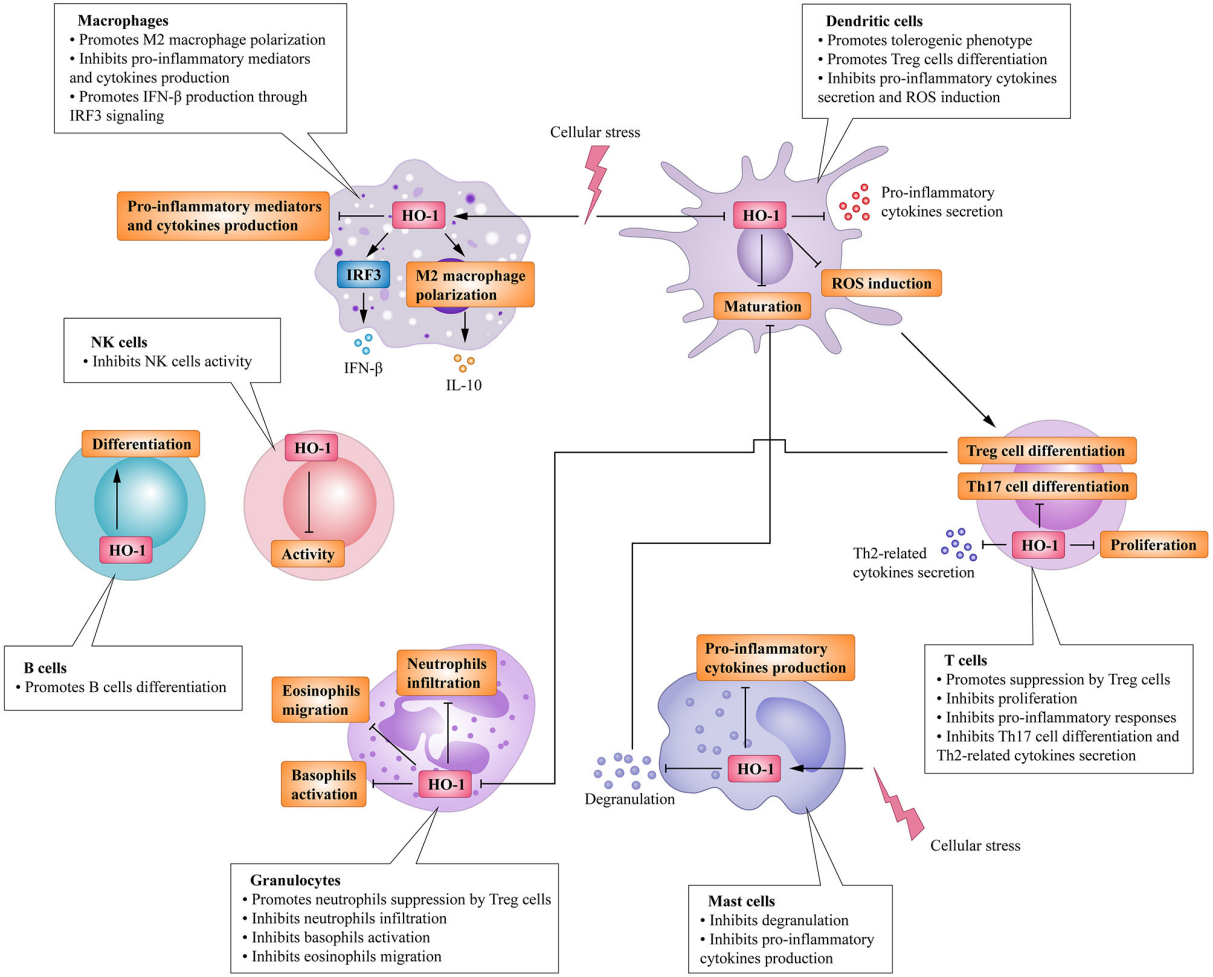
Immunomodulatory activity of heme oxygenase 1 (HO-1) in immune cells. HO-1 is stress-inducible by macrophages and promotes the phenotypic shift to M2 macrophages, associated anti-inflammatory activities. HO-1 also modulates the production of interferon (IFN)-β in macrophages through direct HO-1 binding to interferon regulatory factor 3 (IRF3) in response to pro-inflammatory stimuli. HO-1 induction in dendritic cells (DCs) inhibits their maturation, secretion of pro-inflammatory cytokines, and induction of ROS. Mast cells (MCs) can sense local stressor insults to induce HO-1, thereby suppressing their degranulation and production of inflammatory cytokines. Moreover, inhibiting MCs' degranulation can also inhibit DCs' maturation. These effects all result in the promotion of DCs into a tolerogenic phenotype, thus promoting regulatory T cell (Treg cell) differentiation. HO-1 expression by T cells can additionally inhibit T helper 17 cells (TH17 cells) differentiation, Th2-related cytokines secretion, as well as limit proliferation. Activated Treg cells can initiate HO-1 expression in neutrophils shifting them to a suppressive phenotype, which inhibits their infiltration, basophil activation as well as migration of eosinophils. HO-1 expression by B cells can additionally promote their development and growth. HO-1 induction can also inhibit NK cells effector functions and activity.

Heme oxygenase is a type of microsomal enzyme with anti-oxidant functions, and it is a member of the heat shock protein family (2). As the rate-limiting enzyme of heme catabolism, heme oxygenase decomposes heme to yield carbon monoxide (CO), catalytic iron, and biliverdin (3–5). Biliverdin is subsequently converted to unconjugated bilirubin by biliverdin reductase (3, 4, 6). HO can be subdivided into two subtypes: inducible heme oxygenase-1 (HO-1) and constitutive isoform heme oxygenase-2 (HO-2) (7). Different from HO-2, which is stably expressed in most organs (8), HO-1 can be highly up-regulated by a variety of oxidative stress stimuli to protect organs from the damage of oxidative stress (9). In other words, the induction of HO-1 is considered as an adaptive cellular response against the toxicity of oxidative stress (10). More recently, HO-1 has also been recognized as having significant immunomodulatory properties and anti-inflammatory functions (11). The immunomodulatory effects of HO-1 activity have been found in many immune cells (Figure 1). For example, HO-1 expression is particularly up-regulated in macrophages, where they inhibit the production of inflammatory mediators (12–15). Furthermore, HO-1 modulates the production of interferon-β (IFN) in macrophages and dendritic cells (DCs) through direct HO-1 binding to IFN regulatory factor 3 (IRF3) (16). On the other hand, the induction of HO-1 inhibits pro-inflammatory functions and maintains DCs in an immature-like phenotype to promote tolerogenic DCs (tolDCs), with a consequent reduction in effector T cell responses and a promotion of regulatory T cell (Treg cell) responses (17–22). Subsequent studies have reported that the up-regulation of HO-1 in mast cell (MC) can stabilize MC membranes to reduce the production of inflammatory cytokines, suggesting that HO-1 can suppress DC maturation induced by MC degranulation (23, 24). Moreover, HO-1 inhibits the phosphorylation of signal transducer and activator of transcription 3 (STAT3) in naive CD4^+^ T cells, thereby inhibiting T cell proliferation and T helper 17 cell (Th17 cell) differentiation (25), and decreasing T helper 2 cell (Th2 cell)-related cytokine production (26). Furthermore, HO-1 is a critical transcriptional suppressor for B cell development and growth (27), as well as an inhibitor for natural killer cell (NK cell) effector functions (28). These results demonstrate that HO-1 has anti-inflammatory, anti-oxidant, and immunomodulatory properties, and they also imply the therapeutic potential of HO-1 in inflammatory diseases.

The beneficial effects of HO-1 induction in inflammation have also been associated with the degradation of the heme group (29). Under pathogenic conditions, hemoproteins release the heme group, which is a universal danger-associated molecular pattern (DAMP) (30). Free heme subsequently binds to toll-like receptor 4 (TLR4) (31), which triggers the production of tumor necrosis factor α (TNFα) (32). Moreover, free heme readily promotes lipid peroxidation and induces oxidative stress through the generation of reactive oxygen species (ROS), which damage cellular structures and tissues (33). HO-1 in macrophages has the ability to catalyze the decomposition of heme, thereby reducing TLR4 activation and ROS formation (34). Meanwhile, HO-1 prevents heme-induced pro-inflammatory M1 macrophages from undergoing polarization and drives the phenotypic shift to anti-inflammatory M2 macrophages (35, 36). Moreover, the products of heme degradation, namely, CO and biliverdin, exert anti-inflammatory and anti-oxidant properties, respectively (37). Biliverdin and its metabolite, bilirubin, exhibit suppressive properties on the activation of CD4^+^ T cells (11, 38, 39). On the other hand, CO can selectively decrease the expression of several pro-inflammatory genes and increase the expression of anti-inflammatory interleukins-10 (IL-10) in macrophages (40). In rodent models of inflammation, HO-1 and its metabolites (CO and biliverdin) have been shown to reduce leukocyte rolling, adhesion, and neutrophil infiltration as well as migration of eosinophils to inflammatory sites (41–45).

**Figure 2:**
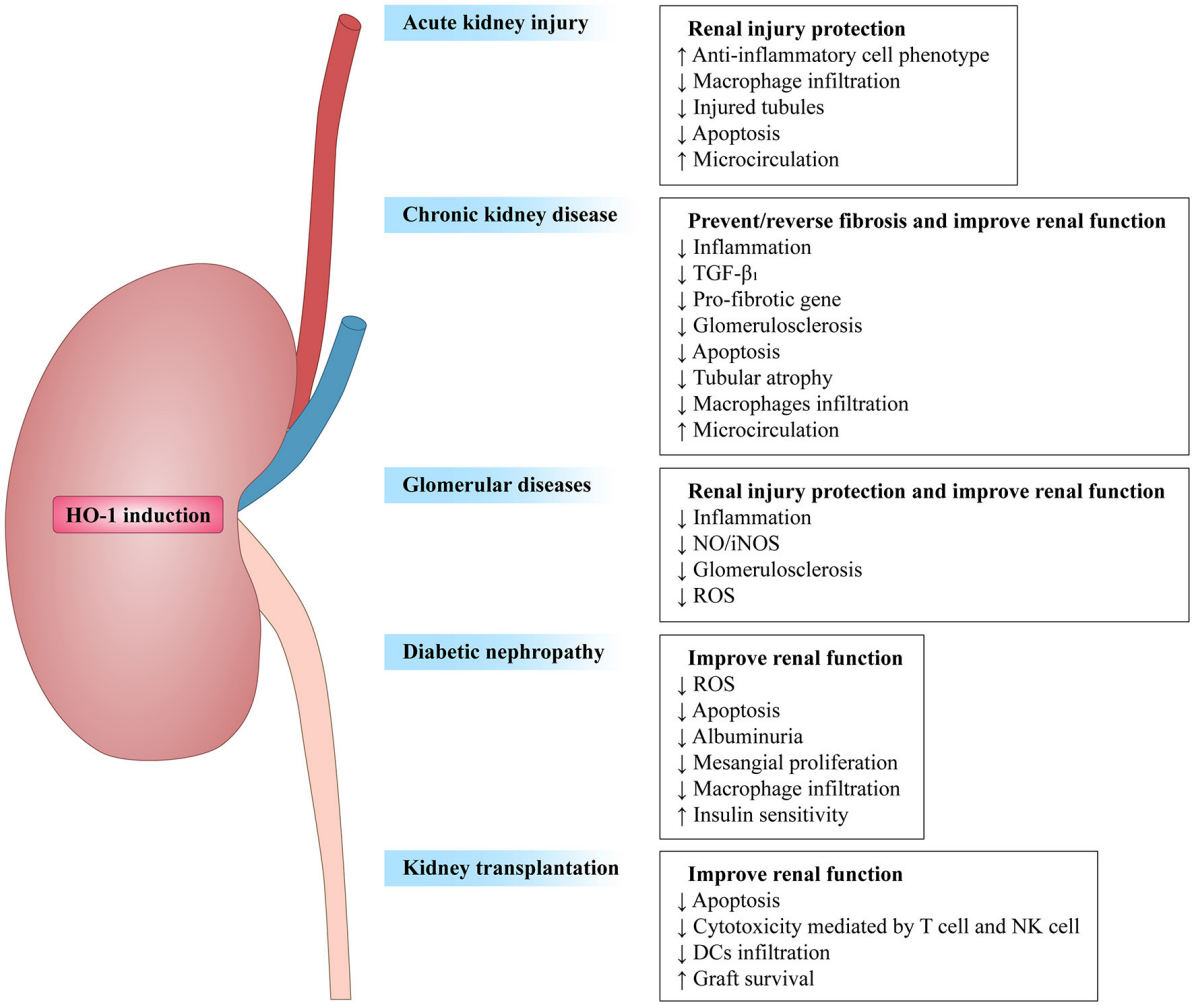
Overview of heme oxygenase-1 in renal diseases. HO-1 induction prevents renal damage in diverse renal diseases, such as acute kidney injury (AKI), chronic kidney disease (CKD), glomerular diseases, diabetic nephropathy (DN), and kidney transplantion. Arrows followed by text indicate increase (↑) or decrease (↓).

Taken collectively, HO-1 activity has been confirmed to affect both innate and adaptive immune responses (Table 1), leading to the reduction of the early inflammatory response and limiting the subsequent tissue damage (5, 55). As a response to inflammatory stimuli and oxidative stress, the expression of HO-1 could activate or inhibit cell-intrinsic pathways in most cell types (20). Therefore, HO-1 is considered as one of the key regulators of the immune system. Here, we focus this review on the influence of HO-1 on kidney diseases in different immune cell populations and kidney resident cells, with a comprehensive understanding of HO-1 and its therapeutic potential in these diseases. Figure 2 summarizes the beneficial effects of HO-1 in these kidney diseases.

**Table 1:** Effects of heme oxygenase-1 on innate and adaptive immune responses based on experiments.

**Immune cells**	**Express levels**	**Species**	**Contributing factor**	**Effects**	**References**
Macrophages	↑	Human	HO-1	Promotes M2 macrophage polarization	(36)
	↑	Mouse	HO-1	Inhibits pro-inflammatory mediators	(12)
		Mouse	CO	Promotes LPS-induced expression of IL-10	(40)
	↑	Mouse	HO-1, CO	Promotes IFN-β production through IRF3 signaling	(16)
Dendritic cells	↑	Rat	HO-1, CO	Inhibits DCs' maturation, pro-inflammatory cytokines secretion and ROS induction	(17)
	↑	Mouse	HO-1	Promotes Treg cells differentiation	(46)
	↓	Mouse	HO-1, CO	Increases intracellular ROS levels, promotes a mature phenotype, impairs phagocytic and endocytic functions, and increases T cell stimulatory capacity	(22)
Mast cells	↑	Rat	HO-1	Inhibits MCs' degranulation and inflammatory cytokines production	(23)
	↑	Mouse	HO-1	Inhibits DCs' maturation indirectly	(24)
Granulocyes	↓	Human	HO-1	Treg cells initiate HO-1 expression in neutrophils, shifting them to a suppressive phenotype.	(47)
	↑	Mouse	HO-1, CO	Inhibits leukocytes rolling, adhesion and neutrophils infiltration	(41)
	↑	Mouse	HO-1	Inhibits eosinophils migration	(42)
		Human	CO	Inhibits basophils activation	(48)
T cells	↓	Human	HO-1	Induces naive CD4^+^ and CD8^+^ T cells activation, proliferation, and maturation	(49)
		Mouse	Bilirubin	Suppresses CD4^+^ T cell reactivity	(38)
		Mouse	Biliverdin	Inhibits T cell proliferation	(39)
	↑	Human	CO, HO-1	Inhibits CD4^+^ T cells proliferation	(50)
	↑	Mouse	HO-1	Inhibits Th17 cell differentiation	(25)
	↑	Mouse	HO-1	Decreases Th2-related cytokines	(26)
	↑	Human	HO-1	Inhibits cytokine release proliferation, and cytotoxicity of other immune cells	(51)
	↑	Human	HO-1	Influences Treg proliferative behavior, but not their suppressive capacity	(52)
	–	Mouse	HO-1	No alteration on the function of Tregs	(53)
	–	Mouse	HO-1	Impairs Treg function indirectly	(18)
B cells	–	Mouse	HO-1	Suppresses B cells development and growth	(27)
NK cells	↑	Mouse	HO-1	Suppresses NK cells effector functions	(28)
	↑	Rat	HO-1	Reduces peripheral NK cell numbers	(54)

## Acute Kidney Injury

Acute kidney injury (AKI), characterized by a rapid increase in serum creatinine and/or a decrease in urine output, is associated with high rates of morbidity and mortality in hospital and outpatient settings (56, 57). Despite the heterogeneity of causes, the response following an acute insult involves similar pathways, including apoptosis, necrosis, and infiltration of inflammatory cells (58). These processes lead to an exaggerated inflammatory response, further aggravating acute tubular necrosis (ATN) and functional derangement in the form of AKI (59). Certain diseases that result in AKI may cause heme release such as intravascular hemolysis or rhabdomyolysis (60). Due to its small molecular weight and hydrophobicity, heme can translocate across the glomerular vascular endothelial cell membrane, subsequently activating TLR4 signaling and other pathways, and mediating the activation of endothelial cells and the production of inflammatory cytokines (61, 62). Furthermore, excessive proteins, such as hemoglobin or myoglobin, can be released into the circulation (63). These processes may lead to severe adenosine triphosphate (ATP) depletion due to excess protein reabsorption, subsequently causing depletion of energy stores and excessive production of ROS (64). High levels of ROS can induce oxidative stress, followed by impaired cellular homeostasis and cell death (65). Under these conditions, inflammatory cells can be locally activated or depleted from hematopoietic organs, thereby leading to an exaggerated inflammatory response (66).

At early stages of AKI, excessive oxidative stress ultimately results in cell death, which accelerates inflammation directly and indirectly, and further increases oxidative stress (67). In this context, HO-1 can be induced in both renal parenchymal cells and tissue resident leukocytes (68, 69). Innate immune cells, such as macrophages, DCs, and neutrophils, are chief participants in the acute insult and recruited to the injured kidneys (70). HO-1 expression in monocytes/macrophages is beneficial in alleviating the inflammatory response of AKI. HO-1-expressing macrophages tend to be polarized toward the M2 phenotype, up-regulate the expression of anti-inflammatory cytokines (IL-10), suppress pro-inflammatory cytokine (TNFα) secretion, and express reparative genes that are beneficial for tissue recovery after AKI (68, 71). A recent study by Hull et al. demonstrated that HO-1 expression by renal DCs could regulate their migration, allowing them to reside in the kidneys where they enhanced recovery and decreased renal fibrosis after ischemia-reperfusion injury (IRI) (68). Furthermore, the authors also found that HO-1 expression in monocytes/macrophages not only accelerates the exit of these cells from the ischemic kidney tissue, but also their migration to extra-renal sites, in turn attenuating their involvement in renal ischemic injury (68). Additionally, HO-1 expression in antigen-presenting cells (e.g., DCs) is required for optimal Treg cell function, which has been suggested to facilitate recovery following AKI (72, 73).

**Figure 3:**
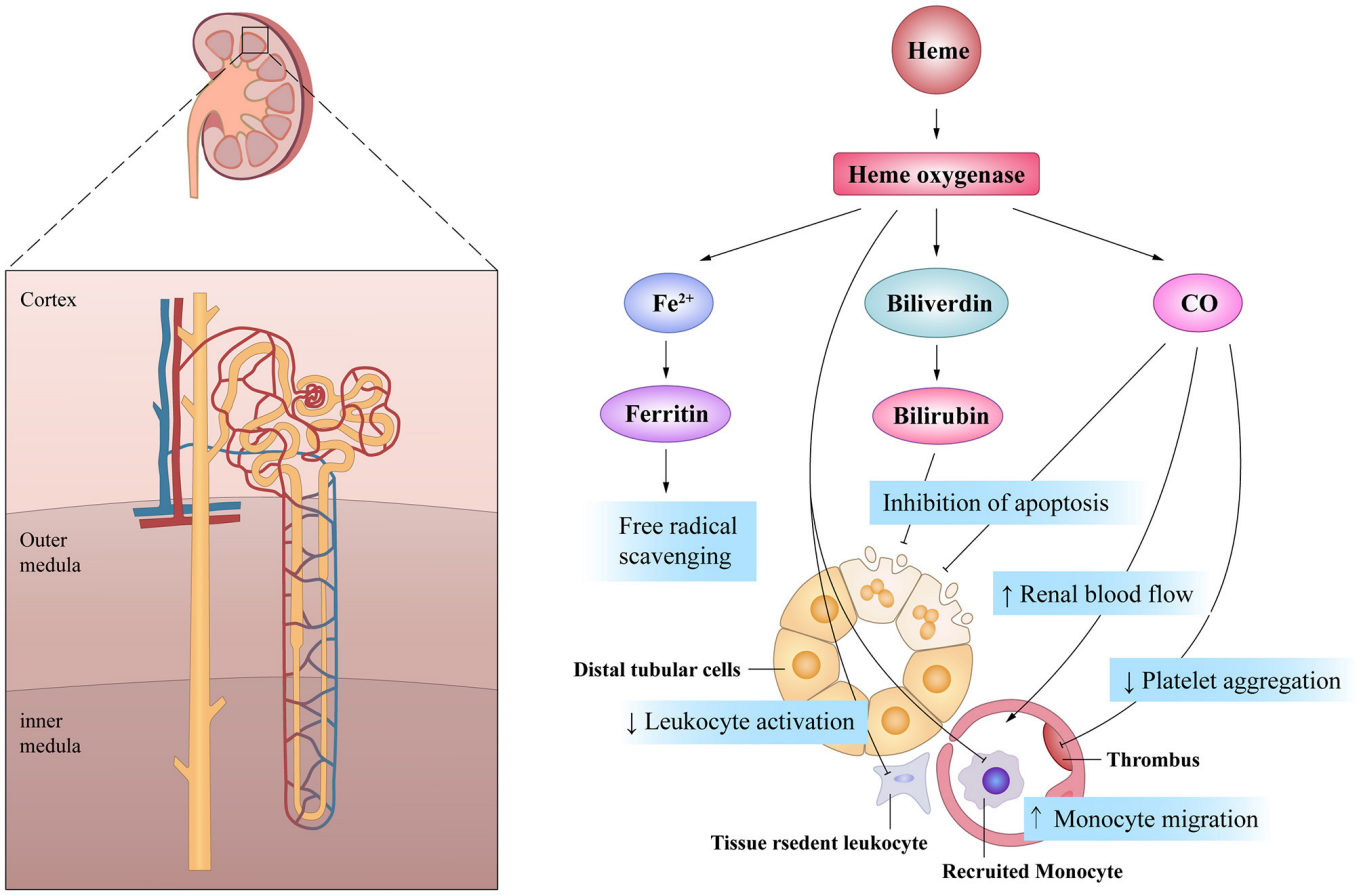
The beneficial actions of HO-1 on renal tubule in acute kidney injury. HO-1 and its products of heme exhibit pleiotropic effects on the cell survival, the inflammatory response and the microcirculation, all of which are of potential importance in mediating its potential protective effects after AKI.

It is worth mentioning that the cytoprotective effects of HO-1 are associated with the by-products of heme degradation. CO exhibits potent anti-proliferative effects on T cells via the down-regulation of IL-2 and caspase activity, which mitigates inflammation (74). Moreover, CO also exerts inhibitory effects on the migration of DCs and promotes immune tolerance (75, 76). CO has significant effects on the circulation by inhibiting platelet aggregation and inducing potent vasodilatory effects (77). Dual-treatment studies with biliverdin and CO in a rat renal transplantation model demonstrated synergistic effects on both blood flow rates and graft survival (78). Furthermore, biliverdin can protect cells from apoptosis induced by cisplatin (CP), a very effective anti-cancer drug, through its anti-oxidative effects (79). CO inhalation therapy can also protect the kidneys from nephrotoxicity induced by CP by limiting renal tubule cell apoptosis (80). Moreover, HO-1 induction is related to the increased availability of ferritin, leading to prompt conjugation and removal of free iron, thereby removing another source of potential oxidative stress (81). Thus, the mechanism of HO-1 may be multifaceted and diversified in terms of the maintenance of renal blood flow and the promotion of cell survival (Figure 3).

The preventive role of HO-1, as well as the induction of other cytoprotective stress proteins, is collectively referred in the literature as “preconditioning treatment” (56). Numerous subsequent studies have confirmed the potential of pharmacologic or genetic HO-1 induction in immune modulation against AKI (71, 82, 83). For instance, statins are capable of exaggerating HO-1 induction post ischemia in vascular smooth muscle cells and macrophages, the administration of which can reduce the severity of IRI-induced AKI, and this protection can be blocked by HO inhibition (83). Moreover, intravenous injection of HO-1-overexpressing macrophages preferentially homes these cells to the ischemic kidney to ameliorate AKI (71). Besides the induction of HO-1 expression under macroscopic conditions, recent studies also have focused on the molecular mechanisms in the induction of HO-1 expression and the regulation of its activity, which includes transcription factors and upstream signaling molecules. For instance, the nuclear factor erythroid 2-related factor 2 (Nrf2) is one of the key transcription factors involved in the regulation of HO-1 (84). During injury, Nrf2 hyperactivation induces the expression of anti-oxidant and detoxification enzymes and downstream proteins, including HO-1 (85). This Nrf2/HO-1 signaling axis can trigger a variety of immunological processes against AKI. Firstly, as one of the catalytic products of HO-1, CO can decrease the activity of nuclear histone acetyltransferase, preventing high-mobility group box 1 (HMGB1) acetylation and release (86), thereby inhibiting the secretion of HMGB1 (87). Recently, studies have shown that HMGB1 activates inflammatory responses by stimulating receptors such as TLR4, and reduces the survival of tubular epithelial cells (86), which promotes renal IR injury (88). Secondly, activation of the Nrf2/HO-1 pathway can reduce the activity of the NACHT, LRR, and PYD domains-containing protein 3 (NLRP3) inflammasome, which is activated by ROS that subsequently decrease the secretion of pro-inflammatory IL-1β and IL-18 (89). Furthermore, T cell-specific augmentation of Nrf2 affects phenotypic diversity, activation, and recruitment of immune cells, which includes increasing anti-inflammatory Tregs, decreasing pro-inflammatory M1 macrophages, and reducing intracellular cytokine production by T cells in ischemic AKI (90). These studies suggest that HO-1, which is induced by the activation of its transcription factor Nrf2, can protect kidneys from the exaggerated inflammatory response during AKI. Given that the multifactorial mechanisms of HO-1 induction are adverse toward the pathophysiological processes of AKI, preventive HO-1 induction may provide new insights that can improve the treatment of AKI.

## Chronic Kidney Disease

Chronic kidney disease (CKD) is a clinical syndrome secondary to the definitive dysfunction and/or the structure of the kidneys and is a public health problem that causes substantial morbidity and mortality (91). In CKD, various abnormally filtered urinary proteins stimulate tubular epithelial cells to synthesize pro-inflammatory mediators, including ROS and chemokines (92). Proteinuria is well-recognized as a prognostic index of the severity of progressive kidney disease and the degree of decline in renal function in CKD (93). Reabsorption of urinary proteins induces ROS production within tubular epithelial cells, which results in the high up-regulation of HO-1 (94). The overexpression of HO-1 in proximal tubular epithelial cells reduces the albumin-stimulated production of cytokines such as monocyte chemoattractant protein-1 (MCP-1) (95). In addition, MCP-1 is a potent chemoattractant, affecting monocytes/macrophages, and increased MCP-1 levels can stimulate transforming growth factor (TGF)-β induction in resident glomerular cells (96). Moreover, MCP-1 induces inflammation by activating nuclear factor-kappaB (NF-κB), thereby directly producing pro-inflammatory effects on the proximal tubules (97, 98). HO-1 knockout mice exhibit drastic interstitial cellular inflammation accompanied by the striking up-regulation of MCP-1 and the activation of NF-κB, which is consistent with previous findings (99).

Theoretically, any etiology that can cause progressive and permanent death of renal tissue and the subsequent replacement of functional nephrons has the potential to lead the fibrosis characteristics of CKD (100). Despite the heterogeneity of etiologies, TGF-β has a critical role in initiating and modulating tissue repair, and its aberrant expression is involved in the pathogenesis of progressive CKD (101). However, this factor can activate the expression of HO-1 to stabilize and attenuate tissue injury (102). Interestingly, a therapeutic approach for HO-1 induction has been proposed in mitigating TGF-β-mediated renal disease (103). In mice with unilateral ureteral obstruction (UUO), a progressive interstitial fibrosis model, the preventive enhancement of HO-1 expression 48 h before UUO attenuated fibrosis by down-regulating inflammatory pro-fibrotic genes as well as pro-apoptotic pathways (caspase-3 activation), which decreased proteinuria and renal dysfunction (104, 105). Zinc protoporphyrin IX (ZnPP), an HO-1 inhibitor, can block these protective effects, thereby inducing increased fibrosis, up-regulating tubular TGF-β1 expression and inflammation, and enhancing the epithelial-to-mesenchymal transition with the increased infiltration of macrophages (106). In transgenic mice, HO-1 overexpression has been reported to limit the tubule-interstitial infiltration of macrophages and to regulate the secretion of inflammatory cytokines, significantly reducing renal interstitial fibrosis in the UUO model (107). Recent evidence indicates that HO-1 can act as a protective agent against renal fibrosis through the regulation of microRNAs, such as the p53-regulated miR-34a and the pro-fibrotic miR-21, but the underlying regulatory mechanisms remain unclear (108). Moreover, administration of low-dose CO to mice has protective effects *via* the MAPK kinase 3 (MKK3) pathway, thereby inhibiting the development of renal fibrosis in obstructive nephropathy (109). These results suggest that the augmentation of HO-1 levels may be a therapeutic strategy against renal interstitial fibrosis.

Interestingly, the level of HO-1 expression is variable across individuals due to the high degree of polymorphism in the number of the guanosine thymidine (GT)_n_ fragment in the promoter (110). This is clinically meaningful, as shown in patients with coronary artery disease in whom a greater number of GT dinucleotide repeats in the HO-1 gene promoter was found to be associated with an increased risk of CKD (111). Thus, dysregulated pathways of inflammation and repair, such as the up-regulated failure of HO-1, may increase oxidative stress and inflammation, which in turn can contribute to this self-injury state (112). Despite the reduced oxygen supply in CKD, hypoxia inducible factors (HIF) are down-regulated and associated with decreased HO-1 expression, although HIF induction was found to restore HO-1 expression in a mouse model of CKD, together with other target genes and angiogenesis (113). Furthermore, Nrf2 activation (and downstream HO-1) may lessen maladaptive repair after repeated acute injuries (114). Thus, the up-regulation of HO-1 expression by Nrf2 or HIF seems to be a potential target for delaying the progression of CKD.

Hemodialysis is one of three renal replacement therapies, and the arteriovenous fistula (AVF) is the preferred hemodialysis vascular access, although it is complicated by high failure rates and attendant morbidity (115). AVF blood flow is markedly reduced in the setting of CKD, and the venous wall is significantly thickened (116). The risk of venous thrombus formation is also increased due to the up-regulation of genes with procoagulant properties (117). However, venous HO-1 induction can improve AVF blood flow and decrease venous wall thickness of the AVF in a murine model of CKD (118). Moreover, the authors found that the administration of carbon monoxide-releasing molecules-3 (CORM-3) (40 mg/kg ip) after nephrectomy and before AVF surgery acutely increases AVF blood flow (118). This study indicates that induction of HO-1 and/or administration of its products may confer beneficial effects in terms of improving certain classical therapies. To conclude, up-regulation of HO-1 during CKD may interrupt the progressive loss of renal function by inhibiting the progression of renal fibrosis. As such, aberrant expression of HO-1 may favor fibrosis by decreasing blood flow, thereby establishing and maintaining a chronic pro-inflammatory state.

## Glomerular Diseases

Glomerular diseases are a leading cause of chronic and end stage renal disease (ESRD) worldwide (119). The unique anatomical location and highly specialized structure of the glomerulus makes the glomerular microvasculature particularly vulnerable, exposes it to a variety of HO-1 inducers. In line with this, up-regulation or induction of HO-1 has been observed in diverse glomerular diseases, including sickle cell nephropathy (120), minimal change disease (121), and IgA nephropathy (121). Interestingly, although the glomerular microvasculature is commonly attacked in these diseases, the above studies demonstrate dominant HO-1 induction primarily in renal tubular rather than glomerular cells (122, 123). This indicates that injury in glomeruli can induce HO-1 expression in renal tubules, which establishes the potential association between glomeruli and tubules.

Glomerulonephritis (GN) refers to a group of renal diseases that attack glomeruli due to damage mediated by immunological mechanisms (124). To study HO-1 expression in acute GN, a model of nephrotoxic nephritis (NTN) has mostly been used (125). In this model, the location of HO-1 expression was also predominantly found in tubular cells and not in glomeruli (126). Under certain circumstances, such as the administration of HO-1 inducer in this model, HO-1 expression can also be induced by glomerulus cells, showing reduced glomerular neutrophils and macrophage infiltration, and decreased glomerular thrombosis (127). In kidney injury, HO-1 expression can be induced in renal parenchymal cells and tissue resident leukocytes (68, 69). These renal tissue resident leukocytes can contribute to lymphocyte differentiation and activation, thereby linking innate and adaptive immune systems (128). HO-1 can also exhibit inhibitory effects on autophagy, which is a highly regulated mechanism to eliminate damaged organelles and proteins from cells and to maintain homeostasis (129).

Inflammatory responses are also regulated by nitric oxide (NO), which is synthesized by nitric oxide synthase (NOS) (130). In GN, intraglomerular inducible nitric oxide synthase (iNOS) activation leads to high levels of NO generation, which results in supraphysiologic amounts of NO within glomeruli (131). The excessive NO can move freely in and out of cells and bind to the heme-iron present in iNOS itself and other hemoproteins (132, 133). Thus, excessive NO can inhibit the subsequent production of NO catalyzed by iNOS (134). Additionally, the binding of NO to heme-containing enzymes can promote destabilization, fragmentation, and proteolysis by proteasomes (135). Thus, NO promotes the release of heme from iNOS, and HO-1 activity is enhanced by the released heme, which can undergo degradation and removal by HO-1 (135). Consistent with these findings, iNOS and HO-1 are co-induced in an NTN model (136). NO production derived from iNOS stimulates HO-1 expression in glomerular mesangial cells, whereas HO-1 activation reduces iNOS expression/activity, which provides evidence for a link between iNOS and HO-1 in NTN rats (136). This may be partly attributable to the expression products of HO-1, all of which can suppress the effects of iNOS expression (137, 138). For instance, CO can bind heme within iNOS and influence the enzymatic activity of iNOS and the production of NO (139). The released iron can suppress iNOS transcription, which down-regulates iNOS expression (140). Another possible reason may be that the heme prosthetic group required by iNOS is degraded by HO-1 (141). Increased HO-1 activity can decrease the availability of heme needed for incorporation into newly synthesized apo-iNOS protein, thereby impairing the synthesis of functional iNOS (142). Moreover, iNOS activity is inhibited when excessive NO binds to heme within the iNOS protein (134). As a result, the activity of iNOS is suppressed and the additional production of NO is blocked, allowing the cells to survive after being exposed to both oxidative and nitrosative stresses. This negative feedback seems to be an excellent mechanism in that glomerular cells can rapidly defend against NO-mediated oxidative injury.

It has also been reported that oxidative stress constitutes a key and a common event in the pathogenesis of IgA nephropathy (143, 144), which is the most common form of primary GN worldwide (145). Nakamura et al. reported that oxidative DNA damage and oxidative stress were increased in patients with IgA nephropathy compared with healthy controls (146). Chen et al. observed that polymorphonuclear leukocyte infiltration, which has a high potential to produce ROS, increased in patients with IgA nephropathy (144). These data suggest that the production of activated ROS is associated with renal dysfunction in IgA nephropathy. ROS are considered to be activated in IgA nephropathy due to the lower mRNA expression of superoxide dismutase (SOD) in moderately or severely damaged tissues from patients with IgA nephropathy and non-IgA mesangial proliferative glomerulonephritis compared with normal or mildly damaged tissues (147). Furthermore, decreased SOD levels may suppress the superoxide-scavenging reaction, thereby rendering the tissues more vulnerable to oxidative stress (147). Accordingly, HO-1 immunoreactivity in the kidneys of patients with IgA nephropathy was significantly higher than that in the kidneys of controls (144). Activated HO-1 can reduce oxidative stress-mediated cellular injury through at least two possible mechanisms, namely, the degradation of cellular heme and the elevation of biliverdin concentrations (148, 149). However, the underlying mechanisms of HO-1 in IgA nephropathy remain unclear, with research pointing to the presence of HO-1 promoter polymorphisms, which predispose individuals to the development of IgA nephropathy (150).

In addition, inflammatory reactions and oxidative stress are involved in the pathogenesis of focal segmental glomerulosclerosis (FSGS), a very common type of chronic kidney disease with clinical features of excessive proteinuria or nephrotic syndrome (151). HO-1-deficient rats exhibit FSGS-type lesions that associate with proteinuria, which implicates HO-1 in the pathobiology of FSGS (152). Aggravated oxidative stress resulting from the absence of HO-1 may be an underlying mechanism that explains the presence of FSGS-type lesions and proteinuria observed in the glomeruli of rats lacking HO-1. Furthermore, several studies have already reported that targeting the Nrf2 anti-oxidant pathway may hold promise as a renoprotective therapy for FSGS (153, 154). Yang et al. observed that targeting the Nrf2-mediated anti-oxidant pathway significantly prevented the development of FSGS in treated mice (153). This delayed effect may involve mechanistic pathways, such as the binding of Nrf2 to anti-oxidant-response elements in the promoter region of several Nrf2-downstream genes encoding anti-oxidant enzymes, including HO-1, glutathione peroxidase (GPx), catalase, and SOD (155, 156). However, it is not entirely clear to what extent HO-1 plays a role in the protection of FSGS.

Taken collectively, inflammatory responses and oxidative stress appear to be a major part of the pathophysiologic process in glomerular diseases. Under these conditions, HO-1 can be induced in tubules, and glomerular injury may initiate HO-1 expression in renal tubules via a potential mechanism. Among them, excessive NO production stimulates HO-1 expression in glomerular mesangial cells, and activation of HO-1 may mitigate NO-mediated toxicity by negatively modulating iNOS expression or activity. HO-1 expression is also upregulated in tissue resident leukocytes. Consequently, glomerular neutrophils and macrophage infiltration are significantly reduced. As an adaptive response to oxidative stress, increased HO-1 expression is also needed to protect cells from oxidative stress, and it may be an emerging therapy in clinical studies. Further studies are required to understand the role of HO-1 in these glomerular diseases.

## Lupus Nephritis

Systemic lupus erythematosus (SLE) is a typical systemic autoimmune disease of unknown etiology that predominantly affects women of child-bearing age (157). It is characterized by chronic inflammation and immunological abnormalities (158). Although inflammation may impact multiple organ systems in SLE, lupus nephritis (LN) remains the representative manifestation and the major contributor to mortality caused by SLE (159). The pro-inflammatory role of monocytes/macrophages in the pathogenesis of SLE has been established. Patients with SLE exhibit a lower level of HO-1 expression in monocytes, suggesting a potential connection between HO-1 expression by myeloid cells and lupus nephritis (160). Cuitino et al. found that the activated neutrophils of patients with LN showed low HO-1 expression, and the baseline ROS level in patients' monocytes was increased, and cobalt protoporphyrin (Co-PP) restored the HO-1 level to the baseline level (161). Thus, the pro-inflammatory environment of LN patients may be associated with decreased HO-1 expression in circulating and infiltrating monocytes/macrophages and neutrophils. However, further studies are needed to determine whether these alterations in immune cells are a cause or a consequence of the disease.

In LN, renal damage is initiated by the production of anti-nuclear antibodies (ANA) and the glomerular deposition of immune complexes (IC) (162). These results are attributed to the failure of T cell and B cell suppression, which is mediated by defects in cell signaling, immune tolerance, and apoptotic mechanisms that promote autoimmunity (163). Accordingly, the administration of tolDCs has been suggested as a potential strategy in the treatment of SLE (164, 165). In a mouse model of SLE, DCs treated *in vitro* with HO-1 inducer showed a stable tolerogenic profile, and treatment with these DCs alleviated SLE symptoms, including decreased ANA and reduced skin lesion severity (166). Likewise, after the administration of hemin as an HO-1 inducer, these mice showed decreased proteinuria, reduced glomerular immune complex deposition, and increased expression of iNOS in the kidneys (167). They also showed decreased circulating levels of anti-dsDNA IgG (a group of ANA) and IFNγ (167). Moreover, CO treatment can also ameliorate proteinuria and renal inflammation in FcγRIIb-deficient mice, a model for SLE (165). CO exposure also significantly decreases the number of activated T cells in the kidneys and lungs, as well as ANA levels in lupus-prone mice (168).

**Figure 4:**
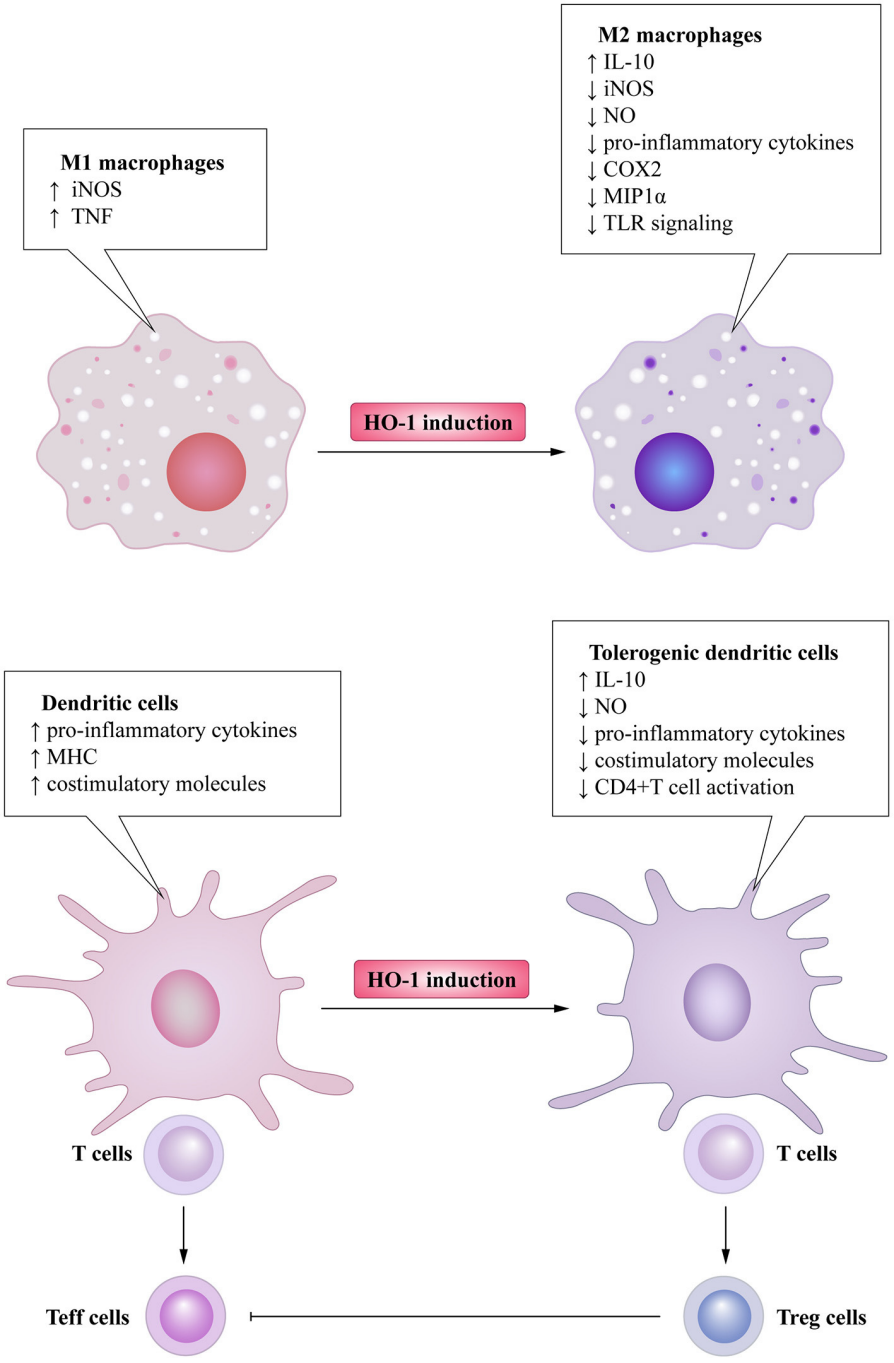
The beneficial effect of the use of HO-1 inducers on the SLE patient. HO-1 induction in immune cells promotes polarization of macrophages into the M2 profile with anti-inflammatory property. They are capable of increasing secretion of IL-10 and decreasing secretion of pro-inflammatory cytokines and mediators. These favor the obtainment of a tolerogenic profile in DCs, thus generating regulatory T (Treg) cells differentiation, which prevents autoimmunity by inhibiting the activation and proliferation of effector T (Teff) cells.

Based on the adverse effects of low HO-1 expression in immune cells, it has become possible to use HO-1 inducers to delay the progression of LN and even ameliorate the systemic conditions of SLE patients (Figure 4). For instance, baicalein can alleviate the symptoms of pristane-induced LN by regulating the balance between Nrf2/HO-1 signaling and NLRP3 expression (169). Furthermore, dietary extra virgin olive oil could attenuate renal damage in a mice SLE model via the activation of the Nrf-2/HO-1 pathway and the reduction of pro-inflammatory cytokines (170). However, further studies are required to provide greater insight into the effects of HO-1 induction and its byproducts.

## Diabetic Nephropathy

Diabetic nephropathy (DN) is one of the most common microvascular complications coinciding with both type I and II diabetes (171). It is characterized by glomerular hypertrophy, proteinuria, decreased glomerular filtration, and renal fibrosis with progressive renal injury caused by high glucose levels (172). About 45% diabetics are affected by this microvascular complication, which ultimately leads to ESRD (173). Chronic hyperglycemia is the major cause of increased ROS production and the leading cause of CKD through the increase of oxidative stress and the induction of cellular damage in patients with diabetes, and thus, it is strongly implicated in the pathogenesis of diabetic-related complications (174). High glucose levels suppress the activation of the HO-1 gene, and reduced HO-1 activity not only increases the levels of heme and superoxide anion (O2^−^) but also decreases the levels of CO and bilirubin, thereby aggravating glucose-mediated oxidative stress (175–178). These observations have been confirmed by experiments with TNF, heme/hemoglobin, and H_2_O_2_ exposure, in which decreased HO-1 activity resulted in reduced cell viability (179–182).

The diabetic db/db mouse model shows increased glomerular HO-1 expression (183). This particular phenomenon was also observed for other animal models of diabetes such as streptozotocin (STZ)-induced diabetes mellitus (184). *In vitro* experiments, high glucose-treated podocytes showed increased HO-1 expression and apoptosis, and the inhibition of HO-1 accentuated podocytes apoptosis (185). Additionally, Nrf2 overexpression in mouse mesangial cells up-regulated the expression of HO-1, as well as reduced high glucose-induced ROS and cell proliferation (186). The reverse effects were observed in cells with Nrf2 knockdown, suggesting a protective role for HO-1 in both podocytes and mesangial cells. Furthermore, HO-1 overexpression prevents apoptosis and cell death mediated by hyperglycemia due to the increased levels of the anti-apoptotic protein B-cell leukemia-lymphoma-xL (Bcl-xL) and several growth factors, as well as the decreased level of MCP-1 (187), again verifying the beneficial effects of HO-1.

Among the HO-1 inducers, hemin can drastically increase HO-1 expression, especially in tubules (188). After chronic administration of hemin in STZ DN rats, these animals showed increased creatinine clearance, decreased iNOS, and decreased urea levels (189). However, hemin can selectively stimulate anti-inflammatory M2 macrophages and IL-10 production, thereby reducing interstitial macrophage infiltration in STZ DN rats (190). Pro-inflammatory M1 macrophages, as well as the suppressive extracellular matrix/profibrotic factors, are also concomitantly abated (190). A recent study has reported that hemin can improve insulin sensitivity by up-regulating HO-1, presumably due to plasma insulin and potentiated agents implicated in insulin sensitization and insulin signaling (191). On the other hand, chromium mesoporphyrin (CrMP), an HO-1 inhibitor, can nullify the hemin-dependent anti-diabetic and insulin-sensitizing effects (191). Several other HO-1 inducers, such as sitagliptin, can mitigate renal injury in STZ-treated rats by activating phosphatidylinositol 3-kinase (PI3K) and Nrf2 (192). Additionally, inducers of HO-1 can improve insulin sensitivity (193), which supports their protective effects and offers the possibility of new therapeutic approaches. Finally, the relevant role of HO-2 in protecting individuals from DN cannot be ignored. STZ-induced diabetes in HO-2-deficient mice stimulates superoxide anion production and provokes prominent tubulointerstitial injury, thereby resulting in enhanced renal dysfunction. Conversely, these negative effects are attenuated when HO-1 is upregulated in these mice (194).

To conclude, chronic hyperglycemia in DN has the capacity to increase oxidative stress and cell apoptosis. In this context, HO-1 can be induced in several types of cells, including podocytes and mesangial cells, thereby limiting podocyte apoptosis and mesangial proliferation. HO-1 also plays an unexpected role in DN under the action of HO-1 inducers by reducing interstitial macrophage infiltration and improving insulin sensitivity.

## Kidney Transplantation

Kidney transplantation is considered to be the mainstay of treatment and the preferred replacement therapy for patients with ESRD (195). The failure of transplanted organs is usually attributed to the early complications of IRI, namely, acute and chronic rejection (196). Although most research has focused on preventing T-cell responses that lead to acute rejection in a more specific and less toxic way, none of these problems have been completely overcome. Transplantation is a state of ischemia-reperfusion, and the main factors affecting organ function after transplantation are nutrient deprivation and hypoxia, with reperfusion aggravating organ damage initially caused by ischemia (197). Oxidative stress, apoptosis, and a non-specific innate immune response that subsequently activates the specific immune system are the leading causes of deteriorated early graft function (198). Accordingly, HO-1 can potentially prevent oxidative stress due to its anti-oxidant and anti-apoptotic properties and suppress the immune response through its immunomodulatory effects.

During organ transplantation, grafts are successively subjected to global cold ischemia, warm ischemia, and blood reperfusion. These steps are thought to compromise graft function and to aggravate both acute and chronic rejection (199). The length of warm ischemia is associated with the extent of tissue damage in renal IRI. The prolonged duration of warm ischemia results in inflammation, local tissue injury, higher free heme levels, and upregulated levels of HO-1, C5a receptor (C5aR), IL-6, and TNF-α (200). However, during the cold ischemia period, high HO-1 expression is related to an inferior outcome, although previous studies cannot identify a direct association between the longer ischemia time and the higher HO-1 expression (201). In a rat model of kidney transplantation, hyperthermic preconditioning or Co-PP induces HO-1 expression. Furthermore, HO-1-induced animals show decreased expression of apoptosis markers and signs of injury, indicating that graft survival and function are improved (202). HO-1 induction by hemin or fenoldopam in donors has also yielded similar beneficial effects that associate with preserved kidney graft function and prevention of apoptosis after reperfusion (203, 204).

T cell and B cell activation by the graft is recognized as the acute rejection episode after kidney transplantation (205). During acute renal allograft rejection, HO-1 is mainly induced in infiltrating macrophages (206). HIF-1 is also significantly up-regulated in both tubules and infiltrating cells, indicating that the rejected grafts are hypoxic (207). This effect may result in the up-regulation of HO-1 in animal models, which may inhibit the cytotoxicity mediated by T cells and NK cells and may reduce the number of DCs in the graft and lymph nodes derived by the donor, and, thus, improve the graft survival (28). Moreover, administration of low doses of cyclosporine A, immune-modulatory peptides, can induce HO-1 to reduce allograft injury and to improve graft function (208). However, recent observations indicate that HO-1 may also be involved in B cell differentiation, which may potentially increase the risk of acute rejection (27).

In a rat chronic renal transplantation model, the induction of HO-1 in the donor kidney by Co-PP improves survival, kidney function, and the morphologic characteristics of grafts (209). The kidneys from Co-PP-treated donor animals exhibit longer preservation under ischemic conditions, and thus, better graft survival (209). Similar findings have also been reported in brain-dead donors, with the up-regulation of HO-1 by Co-PP ameliorating survival of the kidney graft (210). HO-1 overexpression in the recipient can also reduce chronic kidney allograft injury (211). In this regard, there is evidence to suggest that HO-1 can engender cell death induced by the activation of alloreactive T cells, which facilitates graft tolerance (212).

In addition, some of the effects of HO-1 may be associated with the expression of the byproducts of heme degradation such as CO and biliverdin (4, 213, 214). For instance, CO can reduce graft immunogenicity after engraftment and improve allograft function, thereby slowing the progression of chronic allograft nephropathy (76). Despite high doses of CO and their clinical limitations, low doses of CO have been shown to attenuate IRI (207), which was probably due to the stabilization of various enzymes, thereby reducing their degradation and release of heme (215). In a swine model of a kidney allograft, CO exposure reduced acute tubular necrosis and apoptosis, as well as the expression of tissue factor and P-selectin, and enhanced cell proliferative repair (216). Moreover, CO induction before organ procurement may avoid chronic rejection of the kidney in rats (217, 218), and this effect may be more significant with co-administration of CO and biliverdin (78). Furthermore, administration of biliverdin reduced the CD4^+^ T cell response by suppressing immune transcription factor activation, inhibiting co-stimulatory activity, and down-regulating major histocompatibility complex (MHC) class II expression (38). The beneficial effects of bilirubin administration have been proven in patients with islet allografts (219). Thus, HO-1 induction and administration of its products may confer beneficial effects to the transplanted kidney.

## Conclusion

During the past few decades, important roles for HO-1 and its byproducts in the pathophysiology of kidney diseases have been reported and supported by an abundance of evidence. As mentioned above, HO-1 has the capacity to affect the development and the function of a variety of immune cells and kidney resident cells. Under physiological conditions, HO-1 expression in these cells contributes directly or indirectly to the protection of renal function by eliminating free heme and preventing heme-induced inflammation. Under certain pathological conditions, such as kidney transplantation, autoimmune disease, and autoinflammatory disease, HO-1 can regulate and/or inhibit excessive immune responses to cellular stress. HO-1-based immunotherapy may represent a promising strategy to circumvent kidney diseases. These approaches will likely include dietary and herbal medicines or gene therapy-mediated induction of HO-1 as well as the administration of its byproducts. However, it is crucial to consider the pharmacological properties of these compounds as well as the reported discrepancies between *ex vivo*/*in vivo* simulations and the actual clinical situations. The administration of heme and HO-1 byproducts for prolonged periods or at high concentrations exhibits toxic properties. Thus, the enhancement of HO-1 and its byproducts and the down-regulation of free heme should be maintained at acceptable non-toxic levels. Furthermore, despite the amelioration of the underlying disease, chronic HO-1-induced immune suppression remains a significant challenge.

## Author Contributions

YL, KM, and ZH were involved in the conception of the study. YL and KM were involved in writing the article. XS, PZ, ZD, LS, and CL critically revised the manuscript. All authors read and approved the final manuscript.

## Conflict of Interest

The authors declare that the research was conducted in the absence of any commercial or financial relationships that could be construed as a potential conflict of interest.

## Publisher's Note

All claims expressed in this article are solely those of the authors and do not necessarily represent those of their affiliated organizations, or those of the publisher, the editors and the reviewers. Any product that may be evaluated in this article, or claim that may be made by its manufacturer, is not guaranteed or endorsed by the publisher.

## Glossary

HO, heme oxygenase; CO, carbon monoxide; HO-1, heme oxygenase-1; HO-2, heme oxygenase-2; IFN, interferon; DCs, dendritic cells; IRF3, IFN regulatory factor 3; tolDCs, tolerogenic dendritic cells; Treg cells, regulatory T cells; MC, mast cell; STAT3, signal transducer and activator of transcription 3; Th17 cells, T helper 17 cells; Th2 cells, T helper 2 cells; NK cells, natural killer cells; DAMP, danger-associated molecular pattern; TLR4, toll-like receptor 4; TNF, tumor necrosis factor; ROS, reactive oxygen species; IL, interleukins; AKI, acute kidney injury; ATN, acute tubular necrosis; IR, ischemia-reperfusion; ATP, adenosine triphosphate; IRI, ischemia-reperfusion injury; CP, cisplatin; Nrf2, nuclear factor erythroid 2-related factor 2; HMGB1, high-mobility group box 1; NLRP3, NACHT, LRR, and PYD domains-containing protein 3; CKD, chronic kidney disease; MCP-1, monocyte chemoattractant protein-1; TGF, transforming growth factors; NF-κB, nuclear factor-kappaB; UUO, unilateral ureteral obstruction; ZnPP, Zinc protoporphyrin IX; MKK3, MAPK kinase 3; GT, guanosine thymidine; HIF, hypoxia inducible factors; AVF, arteriovenous fistula; CORM-3, carbon monoxide-releasing molecules-3; ESRD, end stage renal disease; GN, glomerulonephritis; NTN, nephrotoxic nephritis; NO, nitric oxide; NOS, nitric oxide synthase; iNOS, inducible nitric oxide synthase; SOD, superoxide dismutase; FSGS, focal segmental glomerulosclerosis; NADPH, nicotinamide adenine dinucleotide phosphate; GPx, glutathione peroxidase; LN, lupus nephritis; SLE, systemic lupus erythematosus; Co-PP, Cobalt Protoporphyrin; ANA, anti-nuclear antibodies; IC, immune complexes; DN, diabetic nephropathy; O_2_^–^, superoxide anion; STZ, streptozotocin; CrMP, chromium mesoporphyrin; PI3K, phosphatidylinositol 3-kinase; Bcl-xL, B-cell leukemia-lymphoma-Xl; C5aR, C5a receptor; MHC, major histocompatibility complex.
